# Turpentine an Excitant of Uterine Contractions

**Published:** 1853-12

**Authors:** Carthon Archer


### *Turpentine an Excitant of Uterine Contractions.*

BY CARTHON ARCHER, M. D.

My attention was first called to the use of the above mentioned agent, as an efficient means of exciting uterine contractions, when deficient, by an article in the London Lancet. It was there stated that when employed in the form of enema, it had uniformly succeeded, even in cases where the ergot had been exhibited without success. Since then, several opportunities have been presented to me of testing its value. In these cases its exhibitions so completely answered my expections as to induce me to submit the following cases, in the hopes of causing a more extensive trial of its efficacy.

The power of ergot in exciting the contractions of the uterus is so well known, that to many it may appear superfluous to seek for new agents to fulfil that indication, There may be however, circumstances in which it might be difficult or impossible to procure ergot, or where it had been unsuccessfully administered, that would render it necessary to resort to other remedies. In such cases, an article, generally so easily obtained as the spirits of turpentine, becomes of great value. There are also cases in which ergot, from the peculiar nature of the contractions it excites, may be inadmissible, while an agent, which merely increases the strength and force of the contractions of the uterus, without altering their kind, would produce the happiest effects. It is in cases of this kind I believe the spirits of turpentine to be equally safe and efficacious. Without further preface, the following cases are submitted:

Case I.—*Oct.* 11, 1851.—I was requested to visit Mrs. ———, a spare, delicate lady, in labor with her second child. The pains had been experienced for two days, but had been slight during the whole time, and had ceased entirely at the time I proceeded to make the usual examinations. The os uteri was dilated and dilatable; the head had descended considerably, and was in the first position, and the membranes were still unruptured. Not having any preparation of ergot with me, I determined to test the virtues of the spirits of turpentine. An ounce of the spirits of turpentine, in a sufficient quantity of gruel to suspend it, was immediately ordered to be thrown up the rectum. Labor pains soon returned, and in a short time the patient was safely delivered of a son. Mother and child both did well.

Case II.—*February,* 1853.—I was called to Catherine, a slave, in labor with her sixth child. She had been in labor for some time, and the membranes had been ruptured in the commencement of labor. Upon making an examination, the foot was found presenting, and the cord prolapsed and pulseless. As the labor pains seemed to be increasing, and the woman doing well, though the child was dead, she was left for the time to the efforts of nature. A few hours after, another examination being made, the labor held out every prospect of terminating without requiring any assistance. The pains, however, soon commenced growing weak and irregular, and the child being found, upon examination, to have made but little progress, an enema of turpentine was ordered, after which the pains rapidly became stronger, and in a short time she was safely delivered, the child of course being dead.

The foregoing cases are submitted without further comment. In conclusion it only remains to remark that the quantity of turpentine recommended was from two to three ounces. In neither of the above cases was more than one ounce administered. In cases where the necessity for delivery was ugent, it might be well to give a larger quantity.—*Stethoscope.*

